# Epithelial expression of TLR4 is modulated in COPD and by steroids, salmeterol and cigarette smoke

**DOI:** 10.1186/1465-9921-8-84

**Published:** 2007-11-22

**Authors:** Ruth E MacRedmond, Catherine M Greene, Delbert R Dorscheid, Noel G McElvaney, Shane J O'Neill

**Affiliations:** 1 Departments of Medicine/Respiratory Research, Royal College of Surgeons in Ireland, Dublin, Ireland; 2 The James Hogg iCAPTURE Centre for Cardiovascular and Pulmonary Research/Critical Care Group, St. Paul's Hospital, University of British Columbia, Vancouver, Canada

## Abstract

The toll-like receptors (TLRs) are a key component of host defense in the respiratory epithelium. Cigarette smoking is associated with increased susceptibility to infection, while COPD is characterised by bacterial colonisation and infective exacerbations. We found reduced TLR4 gene expression in the nasal epithelium of smokers compared with non-smoking controls, while TLR2 expression was unchanged. Severe COPD was associated with reduced TLR4 expression compared to less severe disease, with good correlation between nasal and tracheal expression. We went on to examine the effect of potential modulators of TLR4 expression in respiratory epithelium pertinent to airways disease. Using an airway epithelial cell line, we found a dose-dependent downregulation in TLR4 mRNA and protein expression by stimulation with cigarette smoke extracts. Treatment with the corticosteroids fluticasone and dexamethasone resulted in a dose-dependent reduction in TLR4 mRNA and protein. The functional significance of this effect was demonstrated by impaired IL-8 and HBD2 induction in response to LPS. Stimulation with salmeterol (10^-6 ^M) caused upregulation of TLR4 membrane protein presentation with no upregulation of mRNA, suggesting a post-translational effect. The effect of dexamethasone and salmeterol in combination was additive, with downregulation of TLR4 gene expression, and no change in membrane receptor expression. Modulation of TLR4 in respiratory epithelium may have important implications for airway inflammation and infection in response to inhaled pathogens.

## Introduction

The lung represents the largest epithelial surface in the body and the respiratory epithelial cell represents the body's first interaction with airborne pathogens. As well as providing a physical barrier to entry of micro-organisms, the epithelium is increasingly recognised to play an important role in innate immunity, and can respond to potential pathogens by releasing a variety of effector molecules of the inflammatory response along with anti-microbial peptides [1,2].

TLR4 is critically important in signalling the inflammatory response to Gram-negative bacteria through recognition of LPS, regulating the inducible expression of many cytokines, chemokines, adhesion molecules and acute phase proteins. We have previously shown that LPS signalling via TLR4 induces production of the anti-microbial peptide human beta-defensin 2 (HBD2) [3], which has a broad spectrum of antimicrobial activity, particularly against Gram-negative bacteria, including *Escherichia coli *and *Pseudomonas aeruginosa *and the yeast *Candida albicans *[4].

Chronic Obstructive Pulmonary Disease (COPD) is a condition characterised by progressive airflow limitation punctuated by exacerbations, associated with airway inflammation [5,6]. The role of bacteria in the pathogenesis and acceleration of COPD remains the subject of some debate, but increasing evidence in recent years supports the importance of bacteria in this disease, as a stimulus to chronic inflammation and a cause of exacerbations [7]. Modulation of TLR4 expression in respiratory epithelium could result in an ineffective host response and failure to eradicate potentially pathogenic organisms, leaving the host susceptible to colonisation, chronic inflammation and acute exacerbations.

This study examined the expression of TLR4 and HBD2 in respiratory epithelium in non-smokers and smokers with COPD. The effect of cigarette smoke was replicated *in vitro *by examining TLR4 mRNA and protein expression and quantifying IL-8 expression in airway epithelial cells stimulated with cigarette smoke extracts. The effects of other potential modulators of TLR expression in respiratory epithelium pertinent to COPD, including the long-acting beta_2 _agonist (LABA) salmeterol and the corticosteroids fluticasone and dexamethasone were also examined. The data indicate that altered expression of TLR4 may be important in the pathogenesis of COPD and may be modulated by corticosteroids, LABAs and cigarette smoke.

## Materials and methods

### Study population

Outpatients attending for upper GI endoscopies were recruited for nasal brush sampling following approval of study protocol and consent forms by the Beaumont Hospital Ethics Committee. Subjects were excluded on the basis of pre-existing immunosuppression, pulmonary or nasal pathology, including current or recent (within 6 weeks) upper or lower respiratory tract infection and reported normal functional status.

### Nasal and Tracheobronchial Epithelial cell sampling

Following informed consent, nasal brushing was performed under direct vision using a Cervibrush + (CellPath plc)using a modification of the technique of Bridges et al [8]. Tracheobronchial epithelial cells were harvested as in the method of Kelsen et al [9]. Samples were accepted for analysis if they contained at least 80% epithelial cells.

### Cell lines and culture

Human airway epithelial cells (A549, European Collection of Cell Cultures, Porton Down, UK) were cultured at 37°C in 5% CO_2 _in Ham's F12 (Gibco-BRL), 10% FCS, 1% penicillin/streptomycin. Prior to agonist treatment, cells were washed with serum-free F12 and placed under serum-free conditions or in serum containing 1% FCS for LPS stimulations.

### Preparation of Fluticasone, Salmeterol and Dexamethasone

Fluticasone propionate and salmeterol were obtained from Glaxo SmithKline, Glaxo Wellcome UK Ltd, Stanley Park West, Uxbridge, Middlesex UB11 1BT, and reconstituted Ham's F12/0.01% DMA and Ham's F12/0.01% Methanol respectively to stock concentrations of 10^-6 ^M. Dexamethasone was purchased from Sigma-Aldrich, Tallaght, Dublin, Ireland and reconstituted in 10% Ethanol in PBS to a stock concentration of 1 mM, and serial dilutions prepared in PBS.

### Preparation of cigarette smoke extracts

Cigarette smoke extract (CSE) was freshly prepared for each experiment by a modification of a previously published method [10]. Briefly, 2 filtered Marlboro Red cigarettes, each containing 0.8 mg of nicotine and 10 mg of tar according to the manufacturer's report, were bubbled through 20 ml serum free F-12 medium, pre-warmed to 37°C, by a mechanical vacuum pump. The extract was filtered through a 0.45 μm pore filter (Millipore, Bedford, MA) to remove bacteria and particles, and serial dilutions 1:10 were made.

### Reverse Transcription (RT)-PCR

RNA isolation, cDNA synthesis and RTPCR were performed as previously described [3] using gene-specific primers (Table 1). Products were analyzed by densitometry and compared in a semi quantitative manner relative to GAPDH using ImageMaster^® ^TotalLab Software (Amersham Pharmacia, Amersham, UK).

**Table 1:** 

**Gene (Accession No.)**	**Primers (5'-3')**	**Bases**	**Product Size (bp)**
TLR4 (NM_003266)			
F	AGATGGGGCATATCAGAGC	569-587	481 bp^a^
R	GTCCATCGTTTGGTTCTGG	1068-1050	
TLR4 (NM_003266)*			
F	GGTGGAGCTGTACCGCCTT	2982-3002	65 bp
R	GCCCCAGGACACTGTCCTCCTC	2697-2716	
TLR2 (U 88540)			
F	TGCCCTGCCTATATGCAA	381-398	486 bp
R	GAACACATCGCTGACAACT	936-918	
HBD2 (NM_AF071216)			
F	GGTATAGGCGATCCTGTTACC TGC	2688-2709	202 bp
R	TCATGGCTTTTTGCAGCA TTTTGTTC	4542-4567	
GAPDH (BC004109)			
F	AACTCTGGTAAAGTGGAT	122-138	211 bp
R	TACTCAGCGCCAGCATCG	333-316	

^a ^bp, base pairs, * primers for Real Time PCR

### Real Time PCR

TLR4 mRNA was quantified using commercially available SYBR Green assays as previously described [11] with primers listed in Table 1. The results are expressed as the ratio of the mean of triplicate target gene cDNA measurements to the triplicate housekeeping gene (β-actin) measurement.

### Protein determination

IL-8 protein concentrations in cell supernatants were determined by sandwich ELISA (R & D Systems, U.K.). TLR4 protein was analysed in membrane and cytosolic fractions by Western Blot as previously described [12] and surface expression by Laser Scanning Cytometry as previously described [3].

### Cell viability

Viability of A549 cells under stated treatment conditions was quantified using the Promega CellTiter 96 Aqueous One Solution Cell Proliferation Assay as recommended by the manufacturer.

### Statistical analysis

Data were analyzed with GraphPad Prism 3.0 software package (GraphPad Software, San Diego, CA). Results are expressed as mean ± S.E. and were compared by Mann-Whitney test. Differences were considered significant when the P value was ≤ 0.05.

## Results

### Demographics of patient population

**Figure 1:**
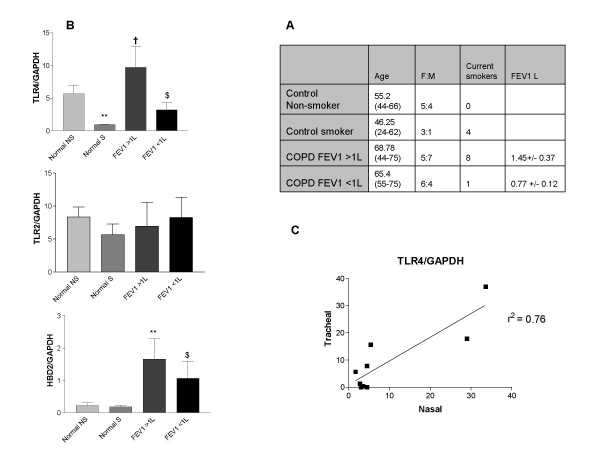
**TLR4 mRNA expression is down-regulated in the nasal mucosa of smokers and in severe COPD *in vivo***. Outpatients attending for upper GI endoscopy or bronchoscopy were recruited for nasal brush sampling. Subjects were excluded on the basis of pre-existing immunosuppression, pulmonary or nasal pathology other than COPD, including current or recent (within 6 weeks) upper or lower respiratory tract infection. Tracheal brush specimens were also collected on a subset of patients undergoing fibreoptic bronchoscopy (n = 9). A. Table showing demographics of the study population. There was no significant difference between the study groups. B. Total RNA from was reverse transcribed into cDNA and used as a template for semi-quantitative PCR reactions using TLR4, TLR2, HBD2 and GAPDH gene-specific primers. (** P < 0.005 vs non-smoking controls; † P < 0.05 vs all controls; $ P < 0.05 vs COPD FEV1 > 1L). C. TLR4 expression by semi-quantitative RTPCR analysis in tracheal and nasal epithelium.

The demographics of the study population are shown in Figure 1A. There was no significant difference in the characteristics of the COPD subgroups or control subgroups in terms of age, gender or medication use. No patients or control subjects reported a clinical history suggestive of atopy. All COPD subjects were using inhaled LABA and corticosteroids. COPD patients were on average a decade older than the control subjects. There was difficulty in recruiting a population of "normal" older smokers, that is, smokers who had no history of respiratory disease and normal FEV1. The main objective of the study was to observe differences between COPD patients of different degrees of severity. Observed differences with control groups represent a "real world" differences between typical subjects with this condition and healthy control subjects. As all COPD patients were using both inhaled LABA and corticosteroids, differences between subsets of patients may be attributable to the disease process, while differences with control subjects may be the result of disease, smoking or medication.

### TLR4 expression is down regulated in the nasal epithelium of smokers in-vivo

We examined expression of TLR4 along with TLR2 and HBD2 in the nasal epithelium of healthy smokers and age matched controls (Figure 1B). Semi-quantitative analysis of mRNA expression revealed a very significant reduction in TLR4 expression in the nasal mucosa of smokers compared to controls (P < 0.005). There was no significant reduction in expression of TLR2 (P = 0.28) or HBD2 (P = 0.20).

### Expression of TLR4 and HBD2 is upregulated in COPD, and decreased in more severe disease

There were no significant differences between mRNA expression of TLR4, TLR2 or HBD2 in nasal epithelium between smokers and non-smokers in either the severe (FEV1 < 1L) or less severe (FEV1 > 1L) COPD (data not shown). Smokers and non-smokers were therefore grouped together for further analysis. As demonstrated in Figure 1B, there was significant upregulation of TLR4 expression in mild to moderate COPD compared to smoking controls († P < 0.05), while severe disease was associated with a significant reduction in TLR4 expression compared to less severe disease (P < 0.05). There was no difference in TLR2 expression between the study groups. Changes in HBD2 expression mirrored those of TLR4, with significant upregulation in mild-moderate COPD compared to controls (P < 0.005), and reduced expression in severe COPD compared to mild-moderate disease (P < 0.05). HBD2 expression in severe COPD was statistically similar to normal controls. (P = 0.17).

### Nasal expression of TLR4 correlated with tracheo-bronchial expression in vivo

In order to see if nasal expression of TLR4 could be extrapolated to expression in the lower respiratory tract, a subgroup of COPD patients underwent bronchoscopy and brush sampling of the tracheo-bronchial epithelium as well as nasal brushing. Data from each of nine subjects is presented in figure 1C, with linear regression analysis demonstrating good correlation between upper and lower respiratory tract expression of TLR4 mRNA (r^2 ^= 0.76, P = 0.001).

### Cigarette smoke condensates down regulate TLR4 expression in respiratory epithelium in-vitro

**Figure 2:**
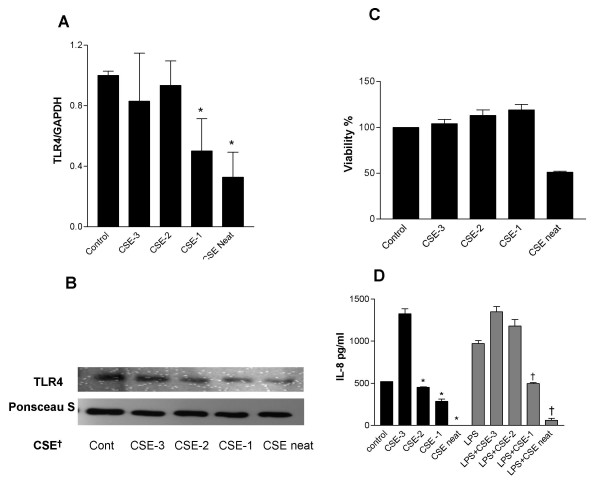
**Cigarette smoke downregulates TLR4 gene and protein expression in A549 cells resulting in relative hyporesponsiveness to LPS**. A549 cells (3 × 10^5^) were seeded onto 6-well plates and grown to confluence. Cells were washed, placed in serum free medium or cigarette smoke condensates for 4 hours. Cigarette smoke condensates were prepared as described in the methods and numbers correspond to serial dilutions of the initial cigarette smoke extract. A. Following treatment, total RNA was extracted, reverse transcribed into cDNA and used as a template for semi-quantitative PCR reactions using TLR4 and GAPDH gene-specific primers. TLR4 expression was given an arbitrary value of 1 in control cells. Data are expressed as mean +/- S.E. and are obtained from three experiments. (* P ≤ 0.05 compared to control). B. Western blot analysis of membrane extracts (10 μg) from A549 cells probed with anti-TLR4 antibody. Data are representative of three separate experiments. (CSE † cigarette smoke extract). Because actin is not compartmentalised to the membrane, equal protein loading is demonstrated with a panel from the Ponsceau Stain. C. Viability assay of A549 cells following treatment with CSE. Data are representative of three separate experiments. D. A549 cells (3 × 10^5^) were seeded onto 6-well plates and grown to confluence. Cells were washed, placed in low-serum (1% FCS) medium and were left untreated or incubated with serial dilutions of CSE × 4 hours. Following treatment with CSE cells were stimulated with LPS 10 *μ*g/ml for a further 24 hours. Levels of IL-8 in supernatants were measured by ELISA and values are expressed as pg/ml. Assays were performed in duplicate a minimum of three times. Values are expressed as mean +/- S.E. (n = 3). (* signifies P ≤ 0.05 of observed effect vs. control, † signifies P ≤ 0.05 of observed effect vs. control plus LPS).

We next examined the effect of cigarette smoke on expression of TLR4 in respiratory epithelium *in vitro*. There was a dose dependant downregulation in TLR4 mRNA (Figure 2A) and protein (Figure 2B) following exposure to the cigarette smoke extracts. To ensure that the effect was not caused by direct toxicity of the cigarette smoke, a viability assay was performed which demonstrated 50% reduction in viability with undiluted CSE, but no toxic effect following dilution of the extracts (Figure 2C) which showed no significant difference in viability compared to untreated cells. We went on to examine functional effect by IL-8 ELISA. As expected, CSE has some direct inflammatory effect resulting in IL-8 production at dilute concentrations. However, concordant with the reduced expression of TLR4, the respiratory epithelial cells have dose dependent reduced secretion of IL-8 following treatment with higher concentrations of CSE, both with and without additional LPS (Figure 2D). Failure of the cells to produce any IL-8 following exposure to undiluted CSE may be a result of the direct toxic effects demonstrated in the viability assay.

### Corticosteroids down regulate TLR4 expression and LPS responsiveness in respiratory epithelial cells

**Figure 3:**
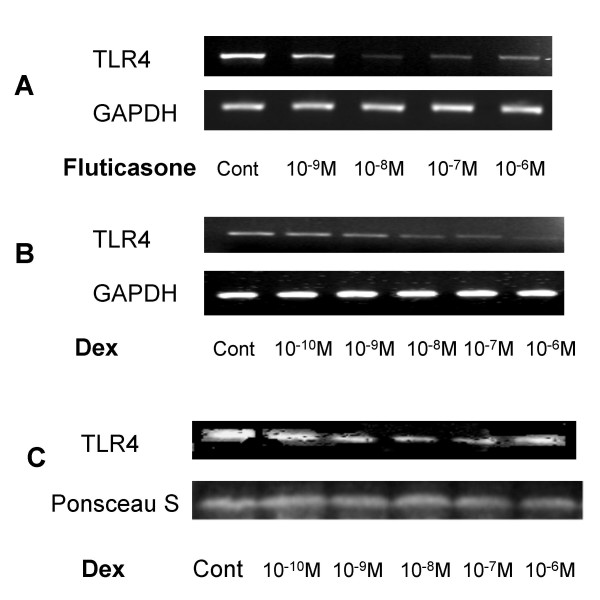
**Corticosteroids downregulate TLR4 expression in respiratory epithelial cells**. A549 cells (3 × 10^5^) were seeded onto 6-well plates and grown to confluence. Cells were washed, placed in low serum (1% FCS) medium and were left untreated or incubated with fluticasone propionate or dexamethasone over the dose ranges 10^-9 ^to 10^-6 ^Molar for 16 hours. A & B. Total RNA was extracted, reverse transcribed into cDNA and used as a template in PCR reactions using, TLR4 and GAPDH gene-specific primers. Products were electrophoresed in 1.5% TBE agarose gels containing 0.5 *μ*g/ml ethidium bromide and visualised under UV. Gels are representative of three independent experiments. C. Western blot analysis of membrane extracts (10 μg) from A549 cells probed with an anti-TLR4 or anti-Actin antibody. Equal protein loading and transfer efficiency was confirmed by Ponceau S staining. Data are representative of three separate experiments.

To explore other potential modulators of TLR4 expression pertinent to COPD, we first examined the effect of Fluticasone on expression of, TLR4 mRNA by RT-PCR in the respiratory epithelial cell line A549 grown in culture (Figure 3A). A dose dependent downregulation of TLR4 compared to the housekeeping gene GAPDH was observed with an Inhibitory Concentration (IC) 50 between 10^-9 ^and 10^-8 ^M. Consistent with the data of Homma et, who found no upregulation of TLR2 in A549 cells treated with dexamethasone alone [13], we found no change in expression of TLR2 or of HBD2 (data not shown).

Fluticasone propionate is a synthetic trifluorinated glucocorticoid. Pharmacologic properties include high lipophilicity, low systemic absorption, rapid metabolism and clearance, and high affinity for the glucocorticoid receptor, resulting in a high therapeutic index as a topical anti-inflammatory agent [14]. Its very low water solubility makes it unpredictable for use in cell culture, however. We therefore assessed whether the observed effect was a class effect of corticosteroids, using the more soluble glucocorticoid dexamethasone. A dose dependent downregulation of TLR4 mRNA (Figure 3B) and protein (Figure 3C) was observed. Consistent with the increased potency of fluticasone, which has approximately 8 times the binding affinity of dexamethasone, a higher dose of dexamethasone was required to achieve a comparable effect (IC_50 _between 10^-8 ^and 10^-7 ^M), whilst acknowledging that these results are semi-quantitative.

**Figure 4:**
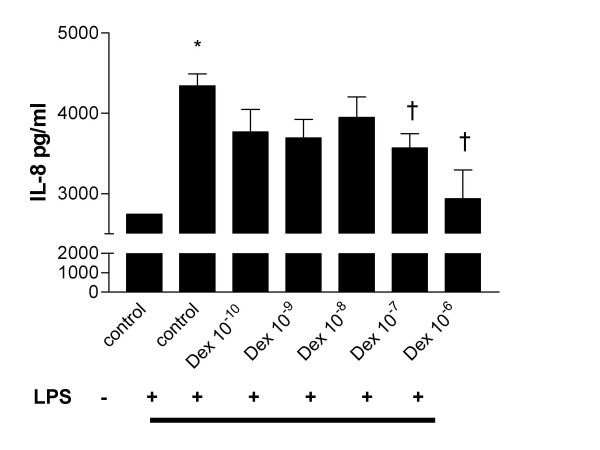
**Downregulation of TLR4 by dexamethasone results in relative hypo-responsiveness to LPS**. A549 cells (3 × 10^5^) were seeded onto 6-well plates and grown to confluence. Cells were washed, placed in low-serum (1% FCS) medium and were left untreated or incubated with dexamethasone at dose of 10^-9 ^to 10^-6 ^Molar for 16 hours. Following treatment with dexamethasone, cells were stimulated with LPS 10 *μ*g/ml for a further 24 hours. Levels of IL-8 in supernatants were measured by ELISA and values are expressed as pg/ml. Assays were performed in duplicate a minimum of three times. Values are expressed as mean +/- S.E. (n = 3). (* signifies P ≤ 0.05 of observed effect vs. control, † signifies P ≤ 0.05 of observed effect vs. control plus LPS).

In order to determine the functional relevance of this effect, we stimulated the cells with the TLR4 agonist LPS. Stimulation of the cells with LPS 10 μg/ml for 24 hours resulted in a significant induction of IL-8, as measured by ELISA of the cell culture supernatant (P < 0.05) (Figure 4). Pre-treatment of the cells with dexamethasone dose-dependently abrogated this effect, reaching statistical significant at a dose of 10^-7 ^M (P < 0.05). A similar effect was seen on the induced expression of HBD2 mRNA in response to LPS (data not shown).

### Membrane expression of TLR4 is upregulated by the long-acting beta-agonist Salmeterol via specific β-agonist effect

**Figure 5:**
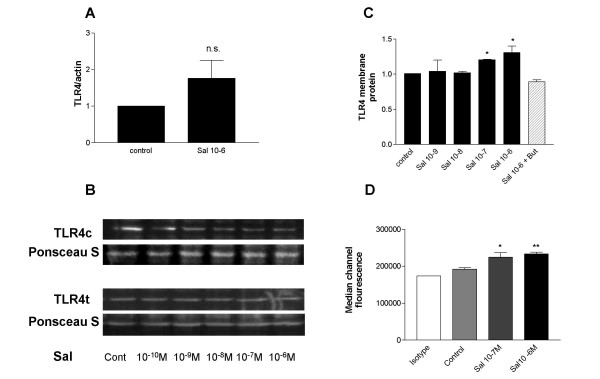
**Salmeterol upregulates TLR4 membrane protein expression in respiratory epithelial cells**. A549 cells (3 × 10^5^) were seeded onto 6-well plates and grown to confluence. Cells were washed, placed in serum free medium and were left untreated or incubated with salmeterol over the dose ranges 10^-9 ^to 10^-6 ^M for 6 hours. Beta-agonist effect was examined by pre-treatment of cells with Butoxamine 0.5 M × 30 minutes prior to salmeterol treatment. A. Real time PCR was performed as described in the methods. Data is expressed as mean +/- SEM of 7 independent experiments with TLR4/actin given an arbitrary value of 1 in control cells. B. Western blot analysis of total cell extracts (t) and cytosolic extracts (c) (10 μg) from A549 cells probed with an anti-TLR4 or anti-Actin antibody. Data are representative of three independent experiments. C. Western blot analysis of membrane extracts (10 μg) from A549 cells probed with an anti-TLR4. Densitometry was performed and corrected for corresponding Ponsceau S staining density. Data are expressed as mean +/- S.E. and are obtained from three experiments. (* P = 0.05 compared to control). D. A459 cells were incubated with an isotype control (clear) or anti-TLR4 (solid) antibody and fluorophore-conjugated detection antibodies. HBD2 expression was quantified by laser scanning cytometry, as described and data from three experiments is presented. HBD2 expression is expressed as Median Channel Fluorescence (MCF) ± SEM. (* P < 0.05 vs. control, ** P < 0.005 vs. control).

We next examined the effect of the long acting beta agonist salmeterol on expression of TLR4 mRNA by RT-PCR over a dose range of 10^-9 ^and 10^-6 ^M. Cells were incubated with the drug for 6 hours. Semi-quantitative analysis suggested a small increase in TLR4 expression over control at the highest dose of 10^-6 ^M, however the lack of a dose response cast doubt on the functional relevance of this observation. We therefore went on to quantify this effect by Real Time RT-PCR and found no significant effect of Salmeterol 10^-6 ^M on TLR4 gene expression (Figure 5A). Analysis of protein expression in total cell lysates similarly showed no significant change in total TLR4 (TLR4t) expression (Figure 5B lower panel), however levels in cytosolic extracts (TLR4c) were decreased (Figure 5B upper panel). A concomitant increase in membrane expression was evident at doses of 10^-7 ^M and 10^-6 ^M. (Figure 5C), an effect which was confirmed by Laser Scanning Cytometry (Figure 5D). Pre-treatment of cells with the beta-blocker Butoxamine abrogated the effect of Salmeterol 10^-6 ^M on membrane expression of TLR4, indicating a specific beta-adrenoreceptor mediated effect (Figure 5C). Taken together these data indicate that salmeterol induces a post-translational transport effect on TLR4.

### Salmeterol reverses the inhibitory effect of dexamethasone on TLR4 expression and LPS responsiveness

**Figure 6:**
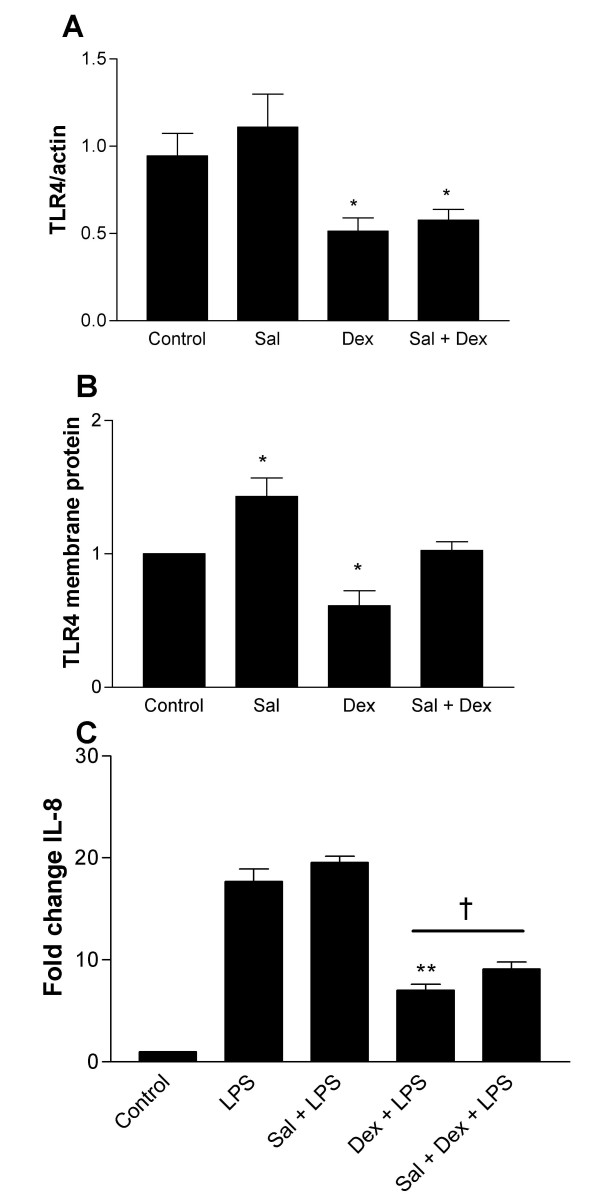
**Salmeterol reverses the inhibitory effect of dexamethasone on TLR4 membrane protein expression despite downregulation of mRNA**. A549 cells (3 × 10^5^) were seeded onto 6-well plates and grown to confluence. Cells were washed, placed in serum free medium and were left untreated or incubated with 10^-6 ^M dexamethasone (Dex), 10^-7^M salmeterol (Sal) or both drugs in combination (Sal + Dex) for 16 hours. Numbers indicate Molar doses of drug. Following treatment, total RNA or membrane protein was extracted for PCR and Western blot analysis. For IL-8 expression analysis, cells were further stimulated with LPS 10 μg/ml × 24 hours. A. Real-time PCR analysis of TLR4 mRNA expression as a factor of β-actin expression. TLR4 expression was given an arbitrary value of 1 in control cells. Data are expressed as mean +/- S.E. and are obtained from three experiments. (* P = 0.05 compared to control). B. Western blot analysis of membrane extracts (10 μg) from A549 cells probed with an anti-TLR4. Densitometry was performed and corrected for corresponding ponsceau staining density. Data are expressed as mean +/- S.E. and are obtained from three experiments. (* P = 0.05 compared to control). C. Levels of IL-8 in supernatants were measured by ELISA and values are expressed as fold change compared to unstimulated control. Assays were performed in duplicate a minimum of three times. Values are expressed as mean +/- S.E. (n = 3). (** signifies P ≤ 0.005 of observed effect vs. LPS alone; † signifies P ≤ 0.05 of observed effect vs. LPS + Dex).

Because inhaled long acting beta agonists are most often prescribed in combination with inhaled corticosteroids, we examined the effect of these compounds used in combination. The lowest dose of dexamethasone at which a functionally significant downregulation of TLR4 was observed, namely 10^-7 ^M, was used in combination with the dose of salmeterol required to produce upregulation of the same receptor, namely 10^-6 ^M. TLR4 gene expression was determined by Real Time PCR (Figure 6A). Again, treatment with salmeterol alone caused no significant change in TLR4 expression above untreated cells, while dexamethasone down regulated TLR4 expression. At the mRNA level, the dexamethasone effect persists when the two compounds are used in combination, resulting in significant downregulation in TLR4 mRNA expression compared to untreated cells. Looking at membrane protein expression however, salmeterol reverses the effect of dexamethasone on TLR4 expression resulting in no net change in TLR4 membrane expression with the two drugs used in combination (Figure 6B). Cell viability was not affected by either drug (data not shown). A similar pattern was observed in LPS-induced IL-8 expression, where the addition of salmeterol partly reversed the impaired IL-8 response to LPS observed with steroid treatment alone (Figure 6C).

### The protective effect of salmeterol is lost in the presence of cigarette smoke extract

**Figure 7:**
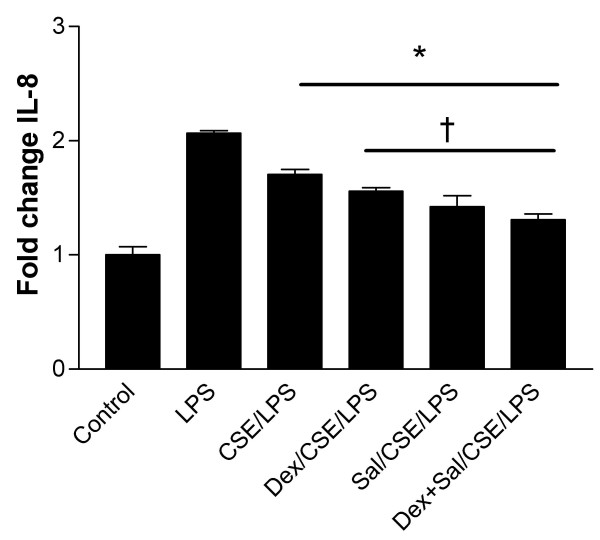
**Cigarette smoke potentiates hyporesponsiveness to LPS by Dexamethasone and Salmeterol**. A549 cells (3 × 10^5^) were seeded onto 6-well plates and grown to confluence. Cells were washed, placed in serum free medium (1 and 2), CSE (10^-1^) × 4 hours [3], pretreated for 16 h with Dex (10^-7 ^M) then for 4 h with CSE (10^-1^) [4], pretreated for 16 h with Sal (10^-6 ^M) then for 4 h with CSE (10^-1^) [5] or pretreated for 16 h with Dex (10^-7 ^M) AND (Sal 10^-6 ^M) then for 4 h with CSE (10^-1 ^M) × 4 hours. Following these treatments, cells were stimulated with LPS 10 *μ*g/ml for a further 24 hours. Levels of IL-8 in supernatants were measured by ELISA and values are expressed as fold change compared to unstimulated controls. Assays were performed in duplicate a minimum of three times. Values are expressed as mean +/- S.E. (n = 3). (* signifies P ≤ 0.05 of observed effect vs. LPS alone, † signifies P ≤ 0.05 of observed effect vs. CSE plus LPS).

As previously demonstrated in figure 2D, IL-8 production in response to LPS was downregulated following exposure to CSE 10^-1^. IL-8 production was further inhibited by pre-treatment with dexamethasone consistent with an additive effect of downregulation of TLR4 expression by both treatments in isolation. Salmeterol treatment was not able to enhance LPS-induced IL-8 expression in the presence of CSE however, and similarly the "protective" effect of salmeterol on dexamethasone-induced inhibition of TLR4 signalling was lost in the presence of CSE. In fact there was further downregulation of IL-8 production (Figure 7). These findings are in keeping with recent report that combination of fluticasone and salmeterol potentiates the suppression of cigarette smoke-induced IL-8 production by macrophages [15]. Salmeterol was found to have no effect on CSE induced IL-8 production in airway smooth muscle cells [16], although the effect of LPS was not examined in these studies.

## Discussion

Expression of TLR4 on respiratory epithelium allows rapid activation of host defense by pathogens, resulting in induction of inflammatory mediators and anti-microbial peptides, including HBD2. Recent evidence also implicates TLR4 deficiency in oxidant induced lung damage and emphysema [17]. Here we report altered expression of TLR4 in the respiratory epithelium of smokers and in patients with COPD, and modifications associated with corticosteroid and LABA treatment that may contribute to our understanding of their therapeutic mechanisms.

Cigarette smoking is a major environmental risk factor predisposing to COPD and is also an independent risk factor for bacterial colonisation of the lower respiratory tract [18,19], acute respiratory infection [20], and infective exacerbations of COPD [21]. Our data demonstrates that smoking is associated with reduced TLR4 expression and LPS responsiveness in respiratory epithelium and is consistent with other data demonstrating reduced HBD2 production in response to LPS in respiratory epithelial cells following exposure to cigarette smoke [22].

TLR4 and HBD2 expression was increased in subjects with mild-moderate COPD compared to normal controls, while with increasing severity of disease and fall in FEV1, expression was reduced. In contrast to alveolar macrophages [23], TLR2 expression is not changed, suggesting that this is not a non-specific response to airway inflammation. There is little existing data regarding the transcriptional regulation of TLRs in human airway epithelial cells, although IFN-γ and TNFα have been shown to modulate TLR4 expression and function in human intestinal epithelium [24,25]. The inflammatory milieu in the airways in COPD includes many potential modulators of TLR4 including cytokines, acute phase reactants [26,27], proteases [28], and anti-proteases [29,30] which may upregulate TLR4 in mild to moderate disease. Whether the reduced expression of TLR4 expression in severe COPD is an adaptive response to increased exposure to Gram-negative pathogens, as part of the phenomenon of endotoxin tolerance [31] in an attempt to attenuate ongoing LPS induced airway inflammation, or pre-exists and thus promotes colonisation [32] is not clear. Reduced epithelial expression of TLR4 may represent a useful biomarker of disease severity.

Our COPD population differed from controls in terms of their exposure to inhaled medications, namely LABAs and corticosteroids. We therefore went on to explore the potential of these compounds to modulate TLR4 expression *in vitro*. Glucocorticoids have been previously reported to modulate lung responses to infection, including Pseudomonas [33]. There have been no previous reports about the effect of corticosteroids on TLR4 expression in epithelial cells. Here we demonstrate that corticosteroid exposure, at clinically relevant doses [34,35] results in downregulation of TLR4 and impaired IL-8 response to LPS. Here we provide evidence for a mechanism whereby corticosteroids could impair host defence against Gram-negative bacteria by downregulation of TLR4 expression.

LABAs such as salmeterol are prescribed primarily as bronchodilators, although accumulating evidence in recent years indicates that LABAs have numerous anti-inflammatory properties [36]. Beta-2 adrenergic receptors are expressed in respiratory epithelium, but the immunomodulatory effect of LABAs on these cells has been largely unexplored. Here we show that the LABA salmeterol had no effect on TLR4 gene transcription or total protein expression, but did induce membrane presentation of TLR4 from the cytoplasmic/nuclear compartment. A similar post-translational effect has been described in nasal epithelium of patients with allergic rhinitis compared to healthy subjects [37], while nuclear localisation of TLR4 has been confirmed in bronchial epithelium [38]. TLR4 has been shown to cycle rapidly between the Golgi and the membrane, with signal transduction occurring only at the membrane [39]. Little is known about the mechanism of this translocation or indeed transport from the nucleus. Our data, demonstrating a beta-receptor mediated effect on post-translational TLR4 transport suggests a potential role for cAMP-dependent protein kinases in this process.

Following in vivo inhalation of 50 μ of salmeterol, the estimated lung tissue concentrations are between 10^-7 ^and 10^-8 ^M [40], and local concentrations at the site of deposition of the drug namely the epithelium are likely higher. The observed effects at doses of 10^-7 ^and 10^-6 ^are therefore clinically relevant.

While the anti-inflammatory effects of corticosteroids are well documented, chronic inhaled corticosteroid therapy alone has failed to impact significantly on disease progression or mortality in numerous large scale multi-centre placebo controlled trials of inhaled corticosteroids in COPD [41-45]. Downregulation of TLR4 membrane protein expression and consequent susceptibility to Gram-negative infection may contribute to the failure of unopposed steroid therapy in these trials. Abrogation of this effect by the addition of salmeterol may represent another important advantage of co-prescription of these compounds, and may contribute to the clinically important improvements in outcome which result when these compounds are prescribed together. In the recent TORCH study, combination therapy with fluticasone and salmeterol resulted in significant reductions in exacerbation rate and 3-year mortality (both COPD related and all cause) compared to fluticasone alone, which had no effect on mortality compared to placebo [46].

In the presence of CSE, the protective effect of salmeterol on TLR4 signalling is lost and in fact there is a small but statistically significant further reduction in LPS-induced IL-8 expression compared to dexamethasone alone. These findings are in keeping with recent report that combination of fluticasone and salmeterol potentiates the suppression of cigarette smoke-induced IL-8 production by macrophages [15]. Although salmeterol was found to have no effect on CSE induced IL-8 production in airway smooth muscle cells [16], although the effect of LPS was not examined in these studies. It would be of great interest to know if the clinical effects of salmeterol and fluticasone in combination were more profound in smokers compared to non-smokers in the TORCH study [46], but this subgroup analysis has not been reported.

The respiratory epithelium is in constant dynamic interaction with the environment, and is uniquely exposed to airborne pathogens and toxins, as well as aerosolised drugs. The TLRs perform a pivotal role in host defence, and this study demonstrates that TLR4 expression in respiratory epithelium is altered in COPD, potentially contributing to the airway inflammation and infective exacerbations which characterise this disease. TLR4 expression is modulated both by drugs used to treat airways inflammation and by cigarette smoke, the major pathogenic determinant of COPD. A greater understanding of the mechanism of these effects may improve our understanding of the pathogenesis of airways disease, and direct future therapies.

## Abbreviations

AMP : Anti-microbial peptide; 

CF : Cystic Fibrosis; 

CSE : Cigarette smoke extract; 

Dex : Dexamethasone; 

EMEM : Eagle's minimal essential medium; 

FCS : Foetal calf serum; 

HBD2 : Human beta-defensin 2; 

IFN-χ : Interferon gamma; 

IL-1β : Interleukin-1 beta; 

IL-8 : Interleukin-8; 

LABA : Long-acting beta_2 _agonist; 

LPS : Lipopolysaccharide; 

NF-κB : Nuclear factor-kappa B; 

Sal : Salmeterol; 

TNFα : Tumour necrosis factor alpha; 

TLR : Toll-like receptor.

## Competing interests

The author(s) declare that they have no competing interests.

## Authors' contributions

RMacR carried out patient recruitment, sample collection and analysis, gene and protein expression analysis, drafted the manuscript and contributed to study design and analysis. CG carried out LSC, cell viability studies and immunoassays, and contributed to manuscript preparation, study design and analysis. DD, NmcE and SON contributed to study design, analysis and manuscript preparation. All authors read and approved the final manuscript.
